# Stress-inducible Protein-1 promotes metastasis of gastric cancer via Wnt/β-catenin signaling pathway

**DOI:** 10.1186/s13046-018-0676-8

**Published:** 2018-01-15

**Authors:** Linlin Huang, Ertao Zhai, Shirong Cai, Yi Lin, Junbin Liao, Huilin Jin, Sui Peng, Lixia Xu, Minhu Chen, Zhirong Zeng

**Affiliations:** 1 Department of Gastroenterology, The First Affiliated Hospital, Sun Yat-sen University, 58 Zhongshan 2nd Road, Guangzhou, 510080 China; 2 Department of Gastrointestinal Surgery, The First Affiliated Hospital, SunYat-sen University, Guangzhou, China; 3 Department of Gastroenterology and Hepatology, Fujian Provincial Hospital, Fuzhou, Fujian China; 4 Department of Hepatobiliary Surgery, The First Affiliated Hospital, Sun Yat-sen University, Guangzhou, China

**Keywords:** STIP1, Metastasis, Epithelial-to-mesenchymal transition, Wnt/β-catenin signaling pathway

## Abstract

**Background:**

Stress-Inducible Protein-1 (STIP1) is a co-chaperone that associates directly with heat shock proteins, and regulates motility of various types of cancer. In the present study, we investigated the role of STIP1 on metastasis of gastric cancer (GC).

**Methods:**

In vivo metastatic experimental model was employed to investigate the effect of STIP1 on metastasis of GC cells. Loss-of-function and gain-of-function experiments were performed to examine the role of STIP1 on metastasis of GC cells. Western blot, immunofluorescence staining, migration and invasion assays, microarray and KEGG pathway analysis were applied to explore the underlying mechanism.

**Results:**

In current study, we demonstrated that STIP1 promoted lung metastasis of GC cells in vivo. Furthermore, STIP1 significantly enhanced migration and invasion abilities of GC cells. In contrast, knock-down of STIP1 yielded the opposite effects on these phenotypes in vitro. STIP1 promoted tumor metastasis through inducing epithelial-to-mesenchymal transition in GC cells. Mechanistically, STIP1 promoted GC metastasis via up-regulation of targeted genes in Wnt/β-catenin signaling pathway, including c-Myc and Cyclin D1, and accompanied with nuclear translocation of β-catenin.

**Conclusions:**

Our findings indicate that elevated expression of STIP1 exhibited a metastasis-promoting effect in GC cells through activation of Wnt/β-catenin signaling pathway. STIP1 may be served as a potential therapeutic target for preventing GC metastasis.

**Electronic supplementary material:**

The online version of this article (10.1186/s13046-018-0676-8) contains supplementary material, which is available to authorized users.

## Background

Gastric cancer (GC) is a common and lethal cancer over the world [1], with marked morbidity and mortality in China [2]. In spite of some developments in the treatment of GC, the overall survival of GC patients remains poor because most GC patients have local and distant metastases at diagnosis [3–5]. Thus, it is of vital importance to investigate the molecular mechanisms underlying metastasis to provide novel biomarkers for treatment of GC.

Tumor growth and metastasis require a series of events within the tumor microenvironment, which involve proliferation, loss of cellular adhesion, degradation of the extracelluar matrix, invasion into host stroma, cell migration, and angiogenesis [6, 7]. Epithelial-to-mesenchymal transition (EMT) is a key event in tumor aggression and metastasis [8]. At molecular level of EMT, cancer cells lose their cell-to-cell contacts by inhibiting the epithelial marker E-cadherin and acquiring the mesenchymal markers like N-cadherin and vimentin [9]. However, the mechanisms and pathways underlying EMT in cancers are not comprehensively understood [10].

Stress-Inducible Protein-1 (STIP1), initially reported as a co-chaperone that associates directly with Hsp70/Hsp90 heat shock proteins, participates in a large number of cellular processes such as RNA splicing, transcription, viral replication, protein folding and translocation, signal transduction, and cell cycle regulation [11]. STIP1 was previously considered to be a protein functioning in intracellular compartments as it lacks transmembrane domain or signal peptide [12]. However, studies indicate that STIP1 could be secreted out of the cells, and then bound to prion protein on cell surface and transduced signals to affect cell proliferation and apoptosis [13–15]. In the last decade, a large number of functions of STIP1 have been reported, which includes the protection of cells in the nervous system, development, cellular maintenance, and tumor proliferation [16]. *STIP1* is located at 11q13, and copy number gain of this region has been found in cancers and linked to poor prognosis [17–20]. STIP1 has been reported to be up-regulated in various types of cancer, including hepatocellular carcinoma [21], pancreatic cancer [22], ovarian cancer [23, 24], colon cancer [25], and cholangiocellular carcimoma [26]. Whether STIP1 involved in the regulation of GC metastasis remains unknown. In the present study, we have sought to investigate the roles of STIP1 on migration and invasion of GC cells through both in vitro and in vivo experiments, and further explored the potential mechanism.

## Methods

### Cell culture

Human gastric cell lines (AGS, BGC823, MGC803, MKN28, MKN45 and SGC7901) were obtained from the Chinese Academy of Science Committee Type Culture Collection Cell Bank (Shanghai, China). All cells lines were cultured in Roswell Park Memorial Institute 1640 (RPMI-1640) medium with supplementation of 10% fetal bovine serum (FBS) (Invitrogen, Carlsbad, CA, USA) and appropriate amounts of penicillin (100 U/ml) and streptomycin (100 mg/ml) in a humidified atmosphere of 5% CO_2_ at 37 °C.

### Western blot

Protein was electrophoretically separated by 10% SDS-PAGE and transferred to PVDF membranes (Millipore, Billerica, MA, USA). The membranes were blocked for 2 h with 5% skim milk in TBST, and incubated with specific primary antibodies (Additional file 1: Table S1) overnight at 4 °C followed by incubation with rabbit or mouse radish peroxidase-coupled secondary antibodies for 2 h. Antibody binding was detected using the enhanced chemiluminescence reagent (Millipore, Billerica, MA, USA).

### Small interfering RNA transfection

Small interfering RNA (siRNA) targeting STIP1 (siSTIP1#1: 5’-GCAAGACTGTCGACCTAAA-3′; siSTIP1#2: 5’-CGATGAAGGACTACACCAA-3′) and a negative control (NC) RNA were synthesized by RiboBio (Guangzhou, China). Cells were seeded in six-well plates and starved overnight, and then transfected with 50 nM siRNA using Lipofectamine 2000 (Invitrogen, Carlsbad, CA, USA) according to the manufacturer’s instructions.

### Plasmid transfection

Cells were seeded in six-well plates and starved overnight, and then pReceiver-120-STIP1 or vector was transfected with Lipofectamine 2000 (Invitrogen, Carlsbad, CA, USA) according to the manufacturer’s instructions.

### Wound scratch assay

Cells were seeded into six-well plates and then cultured to 90% confluence. The confluent cell monolayer was wounded using a sterile 100 μl pipette tip. The suspended cells were washed using normal growth medium. The scratch wound was captured after 24 h and 48 h in three fields. The area of the open wound was quantified using Photoshop (Adobe).

### Migration and invasion assay

Cell migration assays were performed using 24-well transwell chambers (Costar-Corning, New York, USA) with 8.0-μm pore polycarbonate filter. The lower chamber was filled with RPMI-1640 with 10% FBS, and cells (5 × 10^4^ cells/well) serum-starved overnight and pretreated with siRNA or plasmid of STIP1 were added into the upper chamber. After incubation, the non-invading cells were gently removed by scraping with a cotton swab, and the cells migrated to the lower membrane were fixed with methanol, and stained with crystal violet (Beyotime, Nantong, China), photographed, and counted. Cell invasion assay was performed similarly, except that transwell inserts were precoated with matrigel.

### Immunofluorescence (IF) staining

Cells were seeded onto glass cover slips placed in six-well plates, fixed with 4% paraformaldehyde permeabilized in phosphate buffered saline (PBS), which contained 0.1% Triton-X 100, and blocked with 5% bovine serum albumin. The cells were incubated with primary antibodies (Additional file 1: Table S1) at 4 °C overnight. After three times washing with PBS, cells were incubated with secondary antibody for 1 h at room temperature. Cells were stained with DAPI (1μg/mL) for 5 min, and washed in PBS. Images were recorded with a fluorescence microscope.

### Immunohistochemical (IHC) staining

Slides were dried at 37 °C overnight, deparaffinized in xylene, rehydrated through graded alcohol, washed with PBS, immersed in 3% hydrogen peroxide for 15 min to inhibit endogenous peroxidase activity, antigen-retrieved by pressure cooking for 7 min in 10 mM sodium citrate buffer for antigen unmasking (pH 6.0), blocked in normal serum (Vectastain ABC kit), incubated with specific antibodies (Additional file 1: Table S1) overnight in a moist chamber at 4 °C, and incubated with secondary antibodies (Vectastain ABC kit). Slides were stained with 3, 3-diaminobenzidine and counterstained with hematoxylin.

### Construction of stable STIP1 knock-down cells

A short hairpin RNA (shRNA) targeting STIP1 (shSTIP1) and the negative control shRNA were cloned into the pGMLV-SC1 vector. The transduction was performed in SGC7901 cells according to the manufacturer’s recommended protocol. Puromycin (Sigma-Aldrich, St. Louis, MO) was used to select for stably transduced cells.

### RNA extraction and microarray analysis

Total RNA was isolated using RNA plus reagent (TaKaRa, Japan). To identify potential STIP1-targeted genes and related pathways, total RNA from SGC7901- recombinant human STIP1 (SGC7901-rhSTIP1) and SGC7901-IgG cells was analyzed using Illumina microarray (HumanHT-12_V4). Raw data were processed using the manufacturer’s standard protocol and further analyzed using KEGG pathway analysis.

### In vivo metastatic experimental model

For tail vein injection experiment, 1 × 10^6^ cells were injected intravenously through tail vein into the tested nude mice (*n* = 12, 6 for each group). All of the mice were euthanized 6 weeks after injection. The lungs excised from the mice were then fixed in 4% paraformaldehyde and embedded in paraffin, and sections of those tissues were used for histologic study. Tumor nodules that formed on the lung were macroscopically detected.

### Statistical analysis

Data were analyzed for statistical significance using the t test (SPSS Inc., Chicago, IL). In these analyses, *p* value < 0.05 was considered to be significant. All the experiments in vitro had been confirmed for three times.

## Results

### Basic expression level of STIP1 in GC tissues and cells

**Fig. 1:**
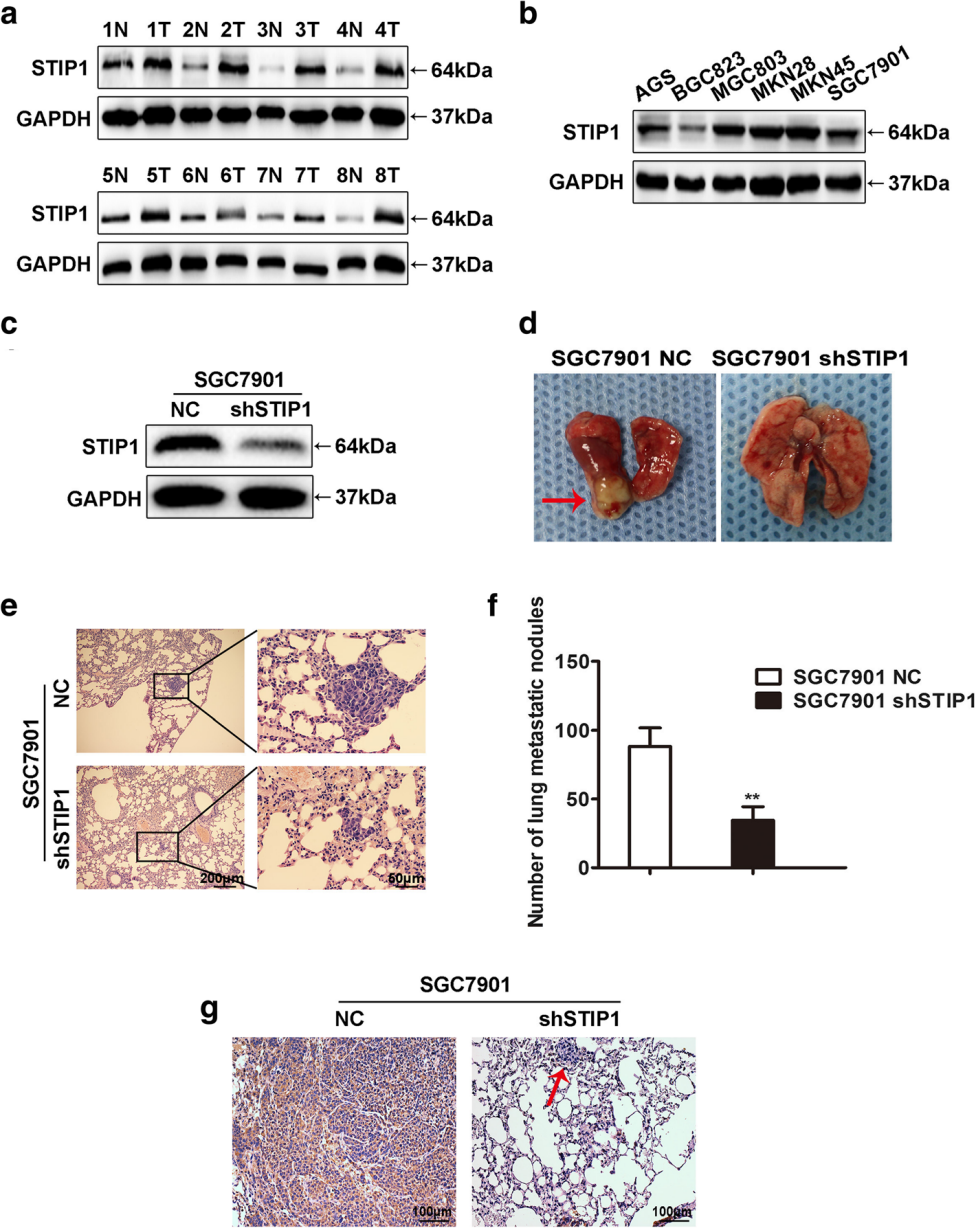
STIP1 promoted lung metastasis of GC cells in vivo. **a** The expression of STIP1 was up-regulating in GC tissues than that in adjacent noncancerous tissues. **b** The basic expression of STIP1 in 6 GC cell lines. **c** The STIP1 expression of SGC7901 NC and SGC7901 shSTIP1 was assessed by Western blot. **d** The representative images of lung metastasis model in nude mice. **e** The representative images of lung metastatic nodules. **f** Compared with control group, mice in SGC7901-shSTIP1 group had significantly fewer lung metastatic nodules. **g** Representative IHC staining of STIP1 expressions in lung of nude mice injected with cells of SGC7901 NC or SGC7901 shSTIP1

Firstly we investigated the expression level of STIP1 in 8 pairs of GC specimens and adjacent noncancerous tissues. The result showed that STIP1 expression was higher in tumors than that in adjacent noncancerous tissues (Fig. 1a), which indicated that STIP1 was up-regulated in GC tissues.

In order to explore the impact of STIP1 on the biological characteristics of GC cells, we assessed expression level of STIP1 in a panel of 6 GC cells (AGS, BGC823, MGC803, MKN28, MKN45 and SGC7901) by Western blot (Fig. 1b). AGS and SGC7901 cells showed moderate STIP1 expression level and therefore were selected for conducting both loss-of-function and gain-of-function experiments.

### STIP1 promoted lung metastasis of GC cells in nude mice model

To investigate the role of STIP1 on GC cells metastasis in vivo, STIP1 stable knock-down cells SGC7901-shSTIP1 and control cells (Fig. 1c) were injected into tail vein of nude mice. 6 weeks later, the mice were sacrificed, and the lung metastatic nodules were counted. Obviously, mice in SGC7901-shSTIP1 group had significantly fewer lung metastatic nodules compared with those in the control group (*p* < 0.01) (Fig. 1d-g), which indicated STIP1 could promote lung metastasis of GC cells.

### STIP1 was associated with migration and invasion of GC cells

To examine the effect of STIP1 on migration and invasion abilities in GC cells, we observed cell morphological changes and carried out wound scratch assay, Transwell assays with/without matrigel in SGC7901 and AGS cells.

**Fig. 2:**
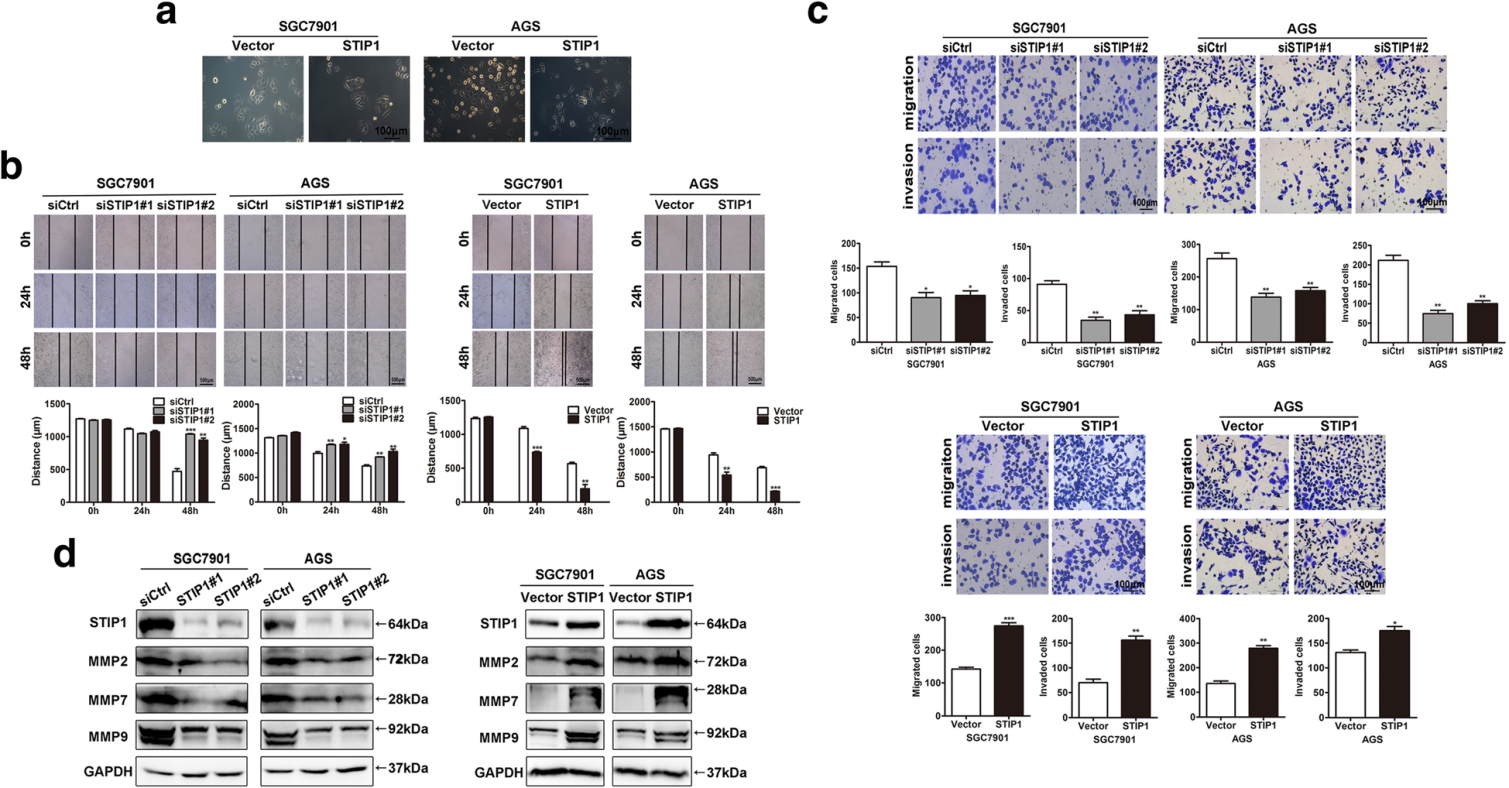
STIP1 promoted cells migration and invasion. **a** SGC7901 and AGS cells were treated with STIP1 or not for 48 h, and the mesenchymal morphological alterations were observed in both cells with overexpression of STIP1. **b** After transfected with siRNA targeted STIP1 (siSTIP1#1, siSTIP1#2), the distance was longer than that in control cells (siCtrl). After overexpression of STIP1, the distance was shorter than that in control cells (Vector). **c** After transfected with siRNA targeted STIP1, the migration and invasion abilities were significantly decreased than that in control cells. After overexpression of STIP1, the migration and invasion abilities were significantly increased than that in control cells. **d** After transfected with siRNA targeted STIP1, the expressions of MMPs (MMP2, MMP7, and MMP9) were reduced than that in control cells. After overexpression of STIP1, the expressions of MMPs (MMP2, MMP7, and MMP9) were elevated than that in control cells. *p < 0.05, **p < 0.01, ***p < 0.001

We first evaluated cellular phenotype with STIP1 overexpression and found that the mesenchymal morphological changes were stimulated in both AGS and SGC7901 cells (Fig. 2a). The wound scratch assay showed that when knock-down of STIP1, the wound healing ability was weaker than that of control cells (all *p* < 0.05) (Fig. 2b). We observed consistent results with STIP1 overexpression (all *p* < 0.05) (Fig. 2b). In order to further validate the migration and invasion abilities of STIP1 in SGC7901 and AGS cells, we performed Transwell assays with/without matrigel. These results confirmed that when STIP1 was knocked down, number of cells transferred to the lower chamber was significantly less than that of control cells (all *p* < 0.05) (Fig. 2c). Consistently, STIP1 overexpression cells had enhanced migration and invasion abilities than those of control cells (all *p* < 0.05) (Fig. 2c). Taken together, these results indicated that STIP1 was associated with migration and invasion of GC cells.

### STIP1 was correlated with matrix metalloproteinases (MMPs)

Based on the fact that MMPs were closely related with migration and invasion abilities of cells, Western blot was applied to determine the MMPs (MMP2, MMP7, and MMP9) expression levels in STIP1 knock-down cells and STIP1 overexpression cells. Protein expression levels of these three MMPs were significantly decreased in STIP1 knock-down group than that in control group (Fig. 2d), while the expression levels of these three MMPs were increased in STIP1 overexpression group than that in control group (Fig. 2d). These data were consistent with the results of migration and invasion experiments in both AGS and SGC7901 cells with different expression levels of STIP1.

### STIP1 was involved in the EMT process

It is well-known that EMT plays an important role in tumor metastasis. Since we found that STIP1 was associated with migration and invasion abilities of GC cells, we further investigated the relationship between STIP1 and EMT.

**Fig. 3:**
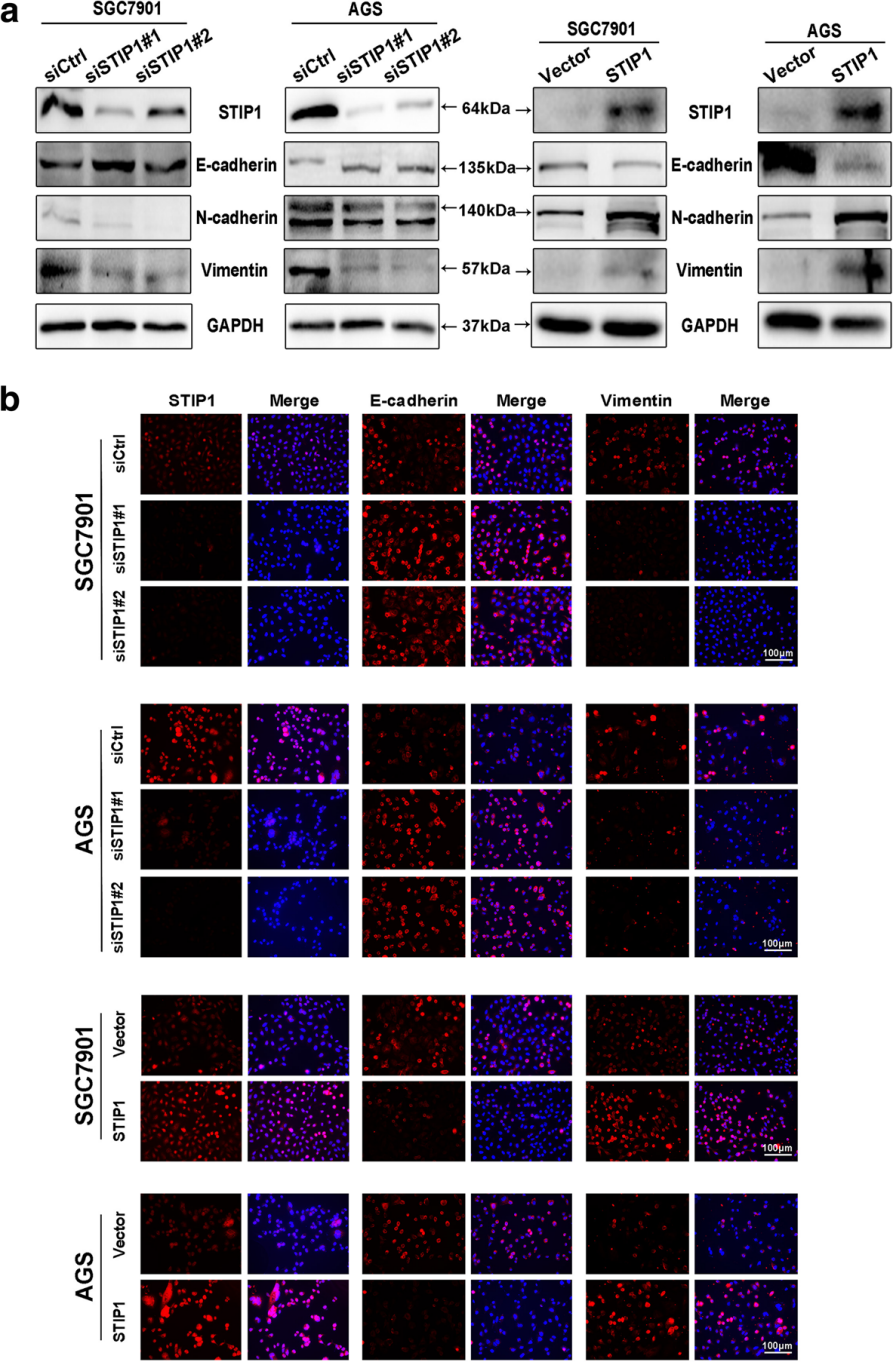
STIP1 was associated with the expressions of EMT markers. **a** Knock-down of STIP1, the expressions of mesenchymal markers (N-cadherin, vimentin) were decreased and the expression of epithelial marker (E-cadherin) was increased by Western blot (Left), while overexpression of STIP1, it yielded the opposite results (Right). **b** Knock-down of STIP1, the mesenchymal marker (vimentin) was decreased and the epithelial marker (E-cadherin) was increased (Upper panel) by IF. In contrary, overexpression of STIP1 yielded the opposite results (Lower panel)

Western blot results showed that the mesenchymal markers (N-cadherin, vimentin) were decreased and the epithelial marker (E-cadherin) was elevated by knock-down of STIP1 (Fig. 3a). Similar results were observed by IF staining in both AGS and SGC7901 cells (Fig. 3b).

Consistently, when STIP1 was overexpressed, mesenchymal markers were upregulated and the epithelial marker was downregulated by both Western blot (Fig. 3a) and IF staining (Fig. 3b).

These data suggested STIP1 was involved in the EMT process to promote metastasis of GC.

### STIP1 promoted metastasis of GC cells via Wnt/β-catenin signaling pathway

**Fig. 4:**
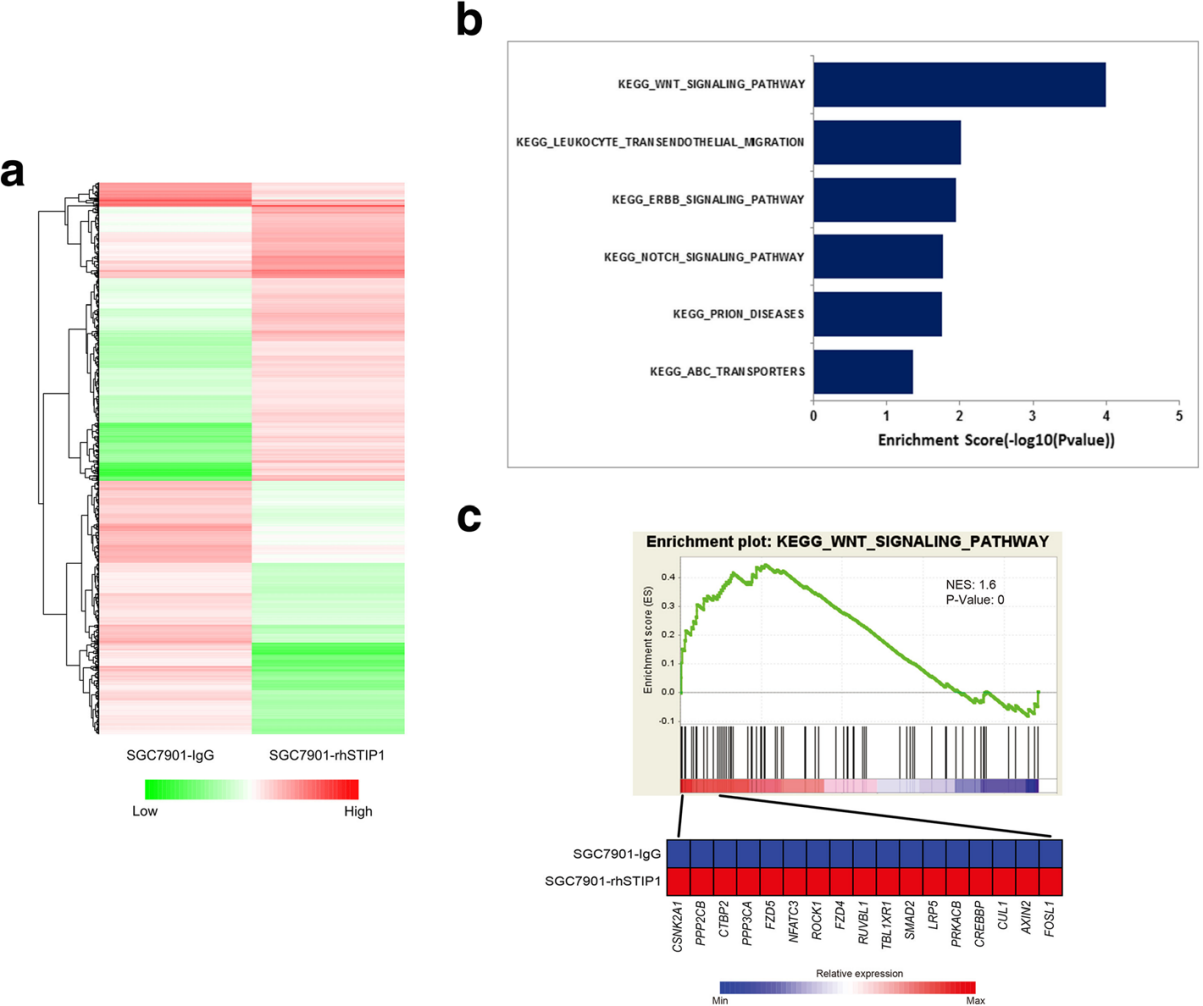
STIP1 was correlated with Wnt signaling pathway. **a** Differentially expressed genes between SGC7901-rhSTIP1 and SGC7901-IgG cells in microarray analyses. **b** KEGG pathway analyses between SGC7901-rhSTIP1 and SGC7901-IgG cells. **c** GSEA revealed that STIP1 significantly correlated with Wnt signaling pathway

To explore the potential mechanism of STIP1 on metastasis of GC cells, we treated SGC7901 cells with rhSTIP1, using IgG as control. Total RNA from SGC7901-rhSTIP1 and SGC7901-IgG cells was analyzed using gene expression microarray. We identified 1683 genes differentially expressed (Fig. 4a). The microarray data were uploaded to Gene Expression Omnibus database and No. is GSE107819.

**Fig. 5:**
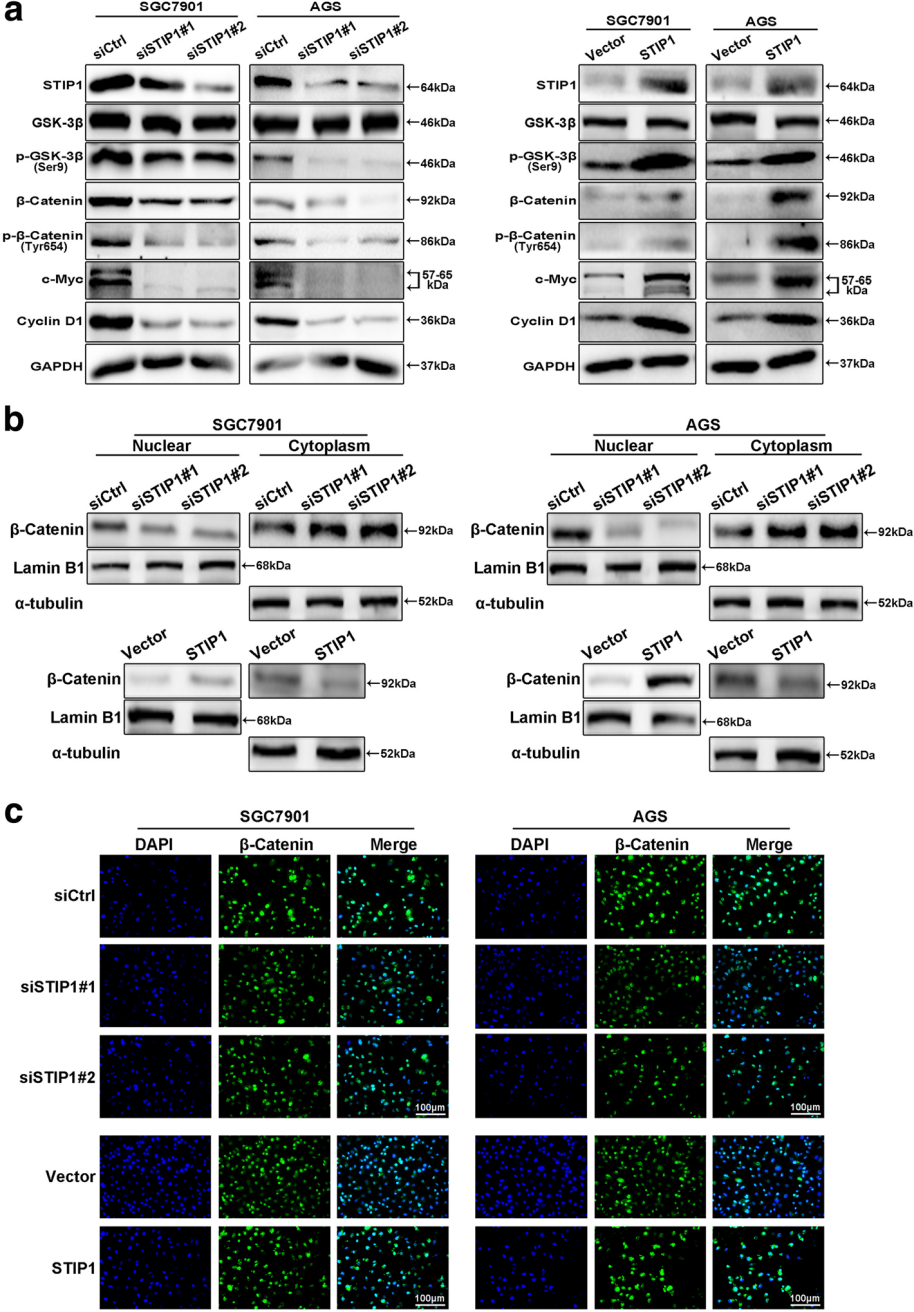
STIP1 was associated with the activation of Wnt/β-catenin signaling pathway. **a** Knock-down of STIP1 markedly reduced the expressions of p-GSK-3β(Ser9), β-catenin, p-β-catenin(Tyr654), c-Myc, and CyclinD1 by Western blot (Left). Overexpression of STIP1 significantly increased the expressions of p-GSK-3β(Ser9), β-catenin, p-β-catenin(Tyr654), c-Myc, and CyclinD1 by Western blot (Right). **b** Knocked down of STIP1 decreased nuclear β-catenin expression and increased cytosolic β-catenin expression by Western blot (Left). Overexpression of STIP1 increased nuclear β-catenin expression and decreased cytosolic β-catenin expression by Western blot (Left). **c** β-catenin nuclear translocation was detected by IF staining

Through KEGG pathway analysis, Wnt/β-catenin signaling pathway was found to be significantly affected (Fig. 4b, c). It is well-known that Wnt/β-catenin signaling pathway cascade is a key pathway in regulating metastasis of GC. To investigate the possible involvement of STIP1 in Wnt/β-catenin signaling pathway, we determined the expression of important genes in Wnt/β-catenin signaling pathway and its targeted genes (such as c-Myc, and CyclinD1). As shown in Fig. 5a, knock-down of STIP1 markedly reduced the expression levels of phosphor-Glycogen synthase kinase-3β (p-GSK-3β)(Ser9), β-catenin, p-β-catenin(Tyr654), c-Myc, and CyclinD1 in both SGC7901 and AGS cells, while overexpression of STIP1 significantly increased the expression levels of p-GSK-3β(Ser9), β-catenin, p-β-catenin(Tyr654), c-Myc, and CyclinD1. These results suggested that STIP1 might play an important role on the activation of Wnt/β-catenin signaling pathway.

As Glycogen synthase kinase-3β (GSK-3β) prevents β-catenin from translocating into the nucleus, we tested the sub-cellular distribution of β-catenin. The results showed that decreased β-catenin was detected in the nucleus of GC cells with knock-down of STIP1, while increased β-catenin was detected in the nucleus of GC cells with overexpression of STIP1 (Fig. 5b). At the same time, expression of β-catenin in cytoplasm had contrary trend (Fig. 5b). To confirm β-catenin translocation, we performed IF staining and found that β-catenin was decreased in nucleus of GC cells with knock-down of STIP1, while β-catenin was increased in nucleus of GC cells with overexpression of STIP1 (Fig. 5c).

**Fig. 6:**
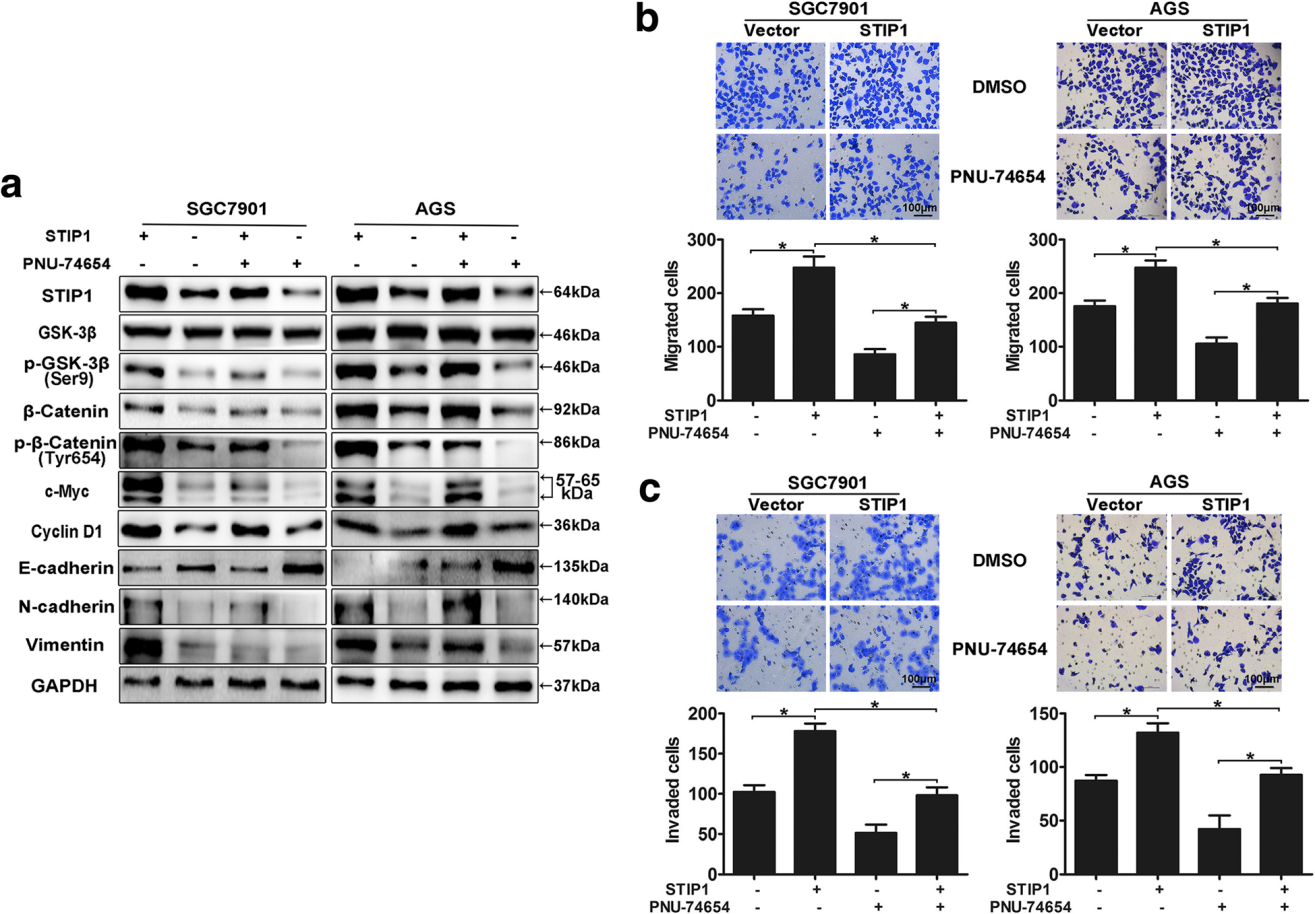
Inhibition of Wnt/β-catenin signaling pathway could not totally abolish STIP1-mediated cells’ migration and invasion abilities. **a** PNU-74654 effectively decreased the expression levels of p-β-catenin(Tyr654), c-Myc, Cyclin D1, as well as N-cadherin and vimentin, and increased the expression level of E-cadherin by Western blot. **b** Treated with/without PNU-74654, the migration number of cells in STIP1-overexpression group was increased than that of control group. **c** Treated with/without PNU-74654, the invasion number of cells in STIP1-overexpression group was increased than that of control group. *p < 0.05

To further determine whether the metastasis-promoting effect of STIP1 was through activation of Wnt/β-catenin pathway, β-catenin inhibitor PNU-74654 was applied. SGC7901 and AGS cells with/without overexpression of STIP1 were treated with/without 50 μmol/L of PNU-74654 for 6 h prior to conduct Western blot, Transwell assays with/without matrigel. The results of Western blot analysis showed that PNU-74654 could effectively decrease the expression levels of p-β-catenin(Tyr654), c-Myc, Cyclin D1, as well as the mesenchymal markers (N-cadherin, and vimentin), and increase the expression level of epithelial marker (E-cadherin) (Fig. 6a). Furthermore, the migration and invasion abilities of STIP1-overexpressed cells could not be totally inhibited by PNU-74654 (all *p* < 0.05) (Fig. 6b, c).

Taken together, these results indicated that STIP1 promoted metastasis of GC cells via Wnt/β-catenin signaling pathway.

## Discussion

It is well known that cell migration and invasion is required for cancer metastasis, which accounts for majority of cancer deaths [27]. Matrix meralloproteinases was reported to play an important role in cell migration and invasion since cell invasion involves degradation of basement membrane extracellular matrix proteins [28]. Previous studies have showed that STIP1 was associated with disease progression and poor prognosis in various types of cancers, particularly for those with advanced lymph node metastasis [21–26]. In this study, we found that STIP1 increased invasiveness and metastatic potential by in vitro and in vivo assays.

Firstly, we observed that STIP1 was up-regulated in GC tissues than that in adjacent noncancerous tissues. From in vivo metastatic experiment, we found that STIP1 could promote GC cells metastasis to lung. We further investigated the relationship between STIP1 and EMT. IF staining results showed that knock-down of STIP1 promoted mesenchymal-to-epithelial transition while overexpression of STIP1 promoted EMT. Moreover, knock-down of STIP1 reduced migration and invasion abilities of AGS and SGC7901 cells, which was associated with decreased expressions of MMP2, MMP7 and MMP9 in both cells. In contrast, overexpression of STIP1 stimulated the migration and invasion of AGS and SGC7901 cells, and increased the expression levels of MMP2, MMP7 and MMP9. Down-regulation of E-cadherin is regarded as one of the critical molecular features involved in the loss of cell-to-cell adhesion, which promotes cancer invasion and metastasis [29]. We observed loss of epithelial cell phenotype and decrease of E-cadherin with STIP1 overexpression. N-cadherin and vimentin are markers of the mesenchymal phenotype. Our study showed that the expression levels of N-cadherin and vimentin were up-regulated in AGS and SGC7901 cells with STIP1 overexpression. These data supported the hypothesis that STIP1 induced EMT in GC cells.

To gain insights into the molecular mechanisms by which STIP1 promoted GC cells migration and invasion, we performed gene expression microarray using rhSTIP1-treated and control SGC7901 cells. The KEGG pathway analysis nominated the Wnt signaling pathway, which was a well-established cell migrated and invaded pathway associated with activation of EMT [30]. GSK-3β was a dual kinase differentially regulated by tyrosine and serine/threonine phosphorylation, and phosphorylation of serine 9 inhibited its kinase activity [31, 32]. β-catenin can be phosphorylated at serine, threonine and tyrosine sites, and phosphorylation of tyrosine 654 promoted translocation of β-catenin from cytoplasm into nucleus, affecting its subcellular location [33–35].

In Wnt/β-catenin signaling pathway, GSK-3β is widely acknowledged for its essential role in phosphorylation, degradation and translocation of β-catenin [36]. Once Wnt/β-catenin signaling pathway is activated, the activity of GSK-3β is inhibited [37]. In our study, we found that overexpression of STIP1 significantly increased the levels of p-GSK-3β (Ser9) and p-β-catenin (Tyr654), and the levels of total β-catenin and nuclear β-catenin were also increased. The expression of c-Myc and Cyclin D1, which are downstream genes of Wnt/β-catenin signaling pathway, were consistent with the expression pattern of β-catenin in STIP1-knockdown and STIP1-overexpression cells. We further confirmed that STIP1 promoted metastasis through the activation of Wnt/β-catenin signaling pathway using β-catenin inhibitor-PNU-74654. Our data demonstrated that PNU-74654 could effectively decrease expressions of key genes of Wnt/β-catenin pathway but could not totally abolish STIP1-medited migratory and invasive ability, implying that the metastasis-promoting function of STIP1 in GC cells was partially dependent on Wnt/β-catenin signaling pathway.

Phosphorylated GSK-3β is a negative regulator of the Wnt/β-catenin activation in gastric cancer. Since STIP1 has no intrinsic phosphatase, it is unlikely that STIP1 directly mediates the phosphorylation of GSK-3β [38, 39]. It is possible that STIP1 regulates the Wnt/β-catenin pathway by indirectly affecting the activity of p-GSK-3β in gastric cancer.

## Conclusions

In summary, we demonstrated that STIP1 promoted cell migration and invasion via Wnt/β-catenin pathway in GC. A better understanding of the oncogenic mechanism of STIP1 in GC may lead to develop novel therapeutic strategy in GC treatment.

## Additional file

Additional file 1: Table S1. Antibodies Used. (DOCX 17 kb)

## Abbreviations

EMT: Epithelial-to-mesenchymal transition
GC: Gastric cancer
GSK-3β: Glycogen synthase kinase-3β
IF: Immunofluorescence
IHC: Immunohistochemical
MMPs: Matrix metalloproteinases
NC: Negative control
PBS: Phosphate buffered saline
p-GSK-3β: Phosphor-Glycogen synthase kinase-3β
SGC7901-rhSTIP1: SGC7901-recombinant human STIP1
shRNA: Short hairpin RNA
shSTIP1: Short hairpin RNA targeting STIP1
siRNA: Small interfering RNA
STIP1: Stress-Inducible Protein-1
