# Supplementary material for: Stress-inducible Protein-1 promotes metastasis of gastric cancer via Wnt/β-catenin signaling pathway
Source: J Exp Clin Cancer Res. 2018 Jan 15;37:6. doi: 10.1186/s13046-018-0676-8 (PMC5769340; doi:10.1186/s13046-018-0676-8)
Supplement: Additional file 1: Table S1. — Antibodies Used. (DOCX 17 kb) [file 13046_2018_676_MOESM1_ESM.docx]

Additional file 1: Table S1. Antibodies Used

| Antibody | Code | Source information | Usage |
| --- | --- | --- | --- |
| STIP1 | #5670 | Cell Signaling Technology | Western blot, IF |
| STIP1 | ab202919 | Abcam | IHC |
| GAPDH | #8884 | Cell Signaling Technology | Western blot |
| MMP2 | #87809 | Cell Signaling Technology | Western blot |
| MMP7 | #71031 | Cell Signaling Technology | Western blot |
| MMP9 | #13667 | Cell Signaling Technology | Western blot |
| E-cadherin | #3195 | Cell Signaling Technology | Western blot, IF |
| N-cadhenrin | #13116 | Cell Signaling Technology | Western blot |
| Vimentin | #5741 | Cell Signaling Technology | Western blot, IF |
| Cyclin D1 | #2978 | Cell Signaling Technology | Western blot |
| c-Myc | #5605 | Cell Signaling Technology | Western blot |
| GSK-3β | #12456 | Cell Signaling Technology | Western blot |
| p-GSK-3β(Ser9) | #5558 | Cell Signaling Technology | Western blot |
| β-catenin | #8480 | Cell Signaling Technology | Western blot, IF |
| Lamin B1 | #13435 | Cell Signaling Technology | Western blot |
| α-tubulin | #3873 | Cell Signaling Technology | Western blot |
| p-β-catenin(Tyr654) | ab59430 | Abcam | Western blot |
